# Roles of follistatin-like protein 3 in human non-tumor pathophysiologies and cancers

**DOI:** 10.3389/fcell.2022.953551

**Published:** 2022-10-17

**Authors:** Shifeng Tian, Xiaoyi Xu, Xiaohui Yang, Linlin Fan, Yuqi Jiao, Minying Zheng, Shiwu Zhang

**Affiliations:** ^1^ Graduate School, Tianjin Medical University, Tianjin, China; ^2^ Department of Stomatology, Tianjin Union Medical Center, Tianjin, China; ^3^ Nankai University School of Medicine, Nankai University, Tianjin, China; ^4^ Graduate School, Tianjin University of Traditional Chinese Medicine, Tianjin, China; ^5^ Department of Pathology, Tianjin Union Medical Center, Tianjin, China

**Keywords:** follistatin-like protein 3, cardiac hypertrophy, myocardial fibrosis, atherosclerosis, reproductive development, stem cell, cancer

## Abstract

Follistatin-like protein 3 (FSTL3) is a type of FSTLs. By interacting with a disintegrin and metalloproteinase 12 (ADAM12), transforming growth factor-β ligands (activin, myostatin and growth differentiation factor (GDF) 11), FSTL3 can either activate or inhibit these molecules in human non-tumor pathophysiologies and cancers. The FSTL3 gene was initially discovered in patients with in B-cell chronic lymphocytic leukemia, and subsequent studies have shown that the FSTL3 protein is associated with reproductive development, insulin resistance, and hematopoiesis. FSTL3 reportedly contributes to the development and progression of many cancers by promoting tumor metastasis, facilitating angiogenesis, and inducing stem cell differentiation. This review summarizes the current pathophysiological roles of FSTL3, which may be a putative prognostic biomarker for various diseases and serve as a potential therapeutic target.

## Introduction

**FIGURE 1:**
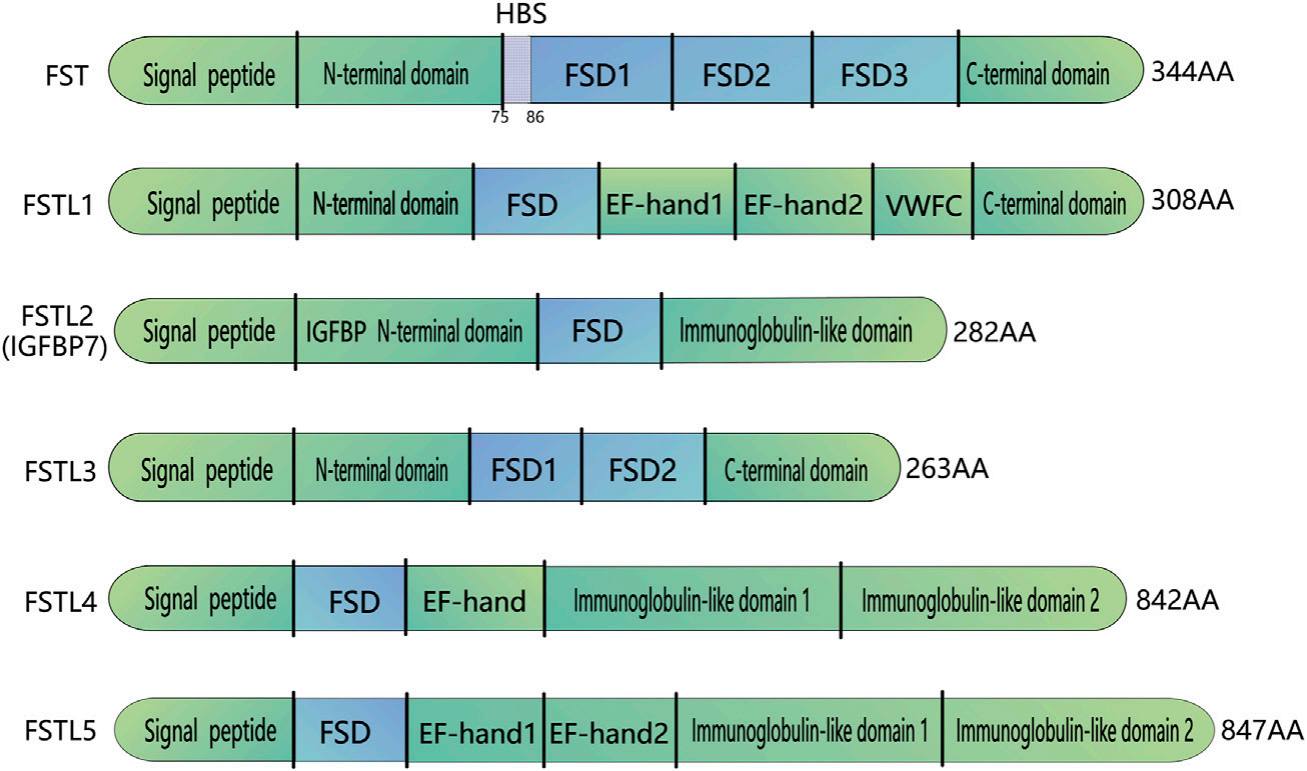
Domain organization of FST and FST-like proteins. FST-like proteins comprise FSTL1, FSTL2, FSTL3,FSTL4, FSTL5. All FST and FST-like proteins contain at least one FSD follistatin domain. FSTL3 does not have FSD3 and the HBS region located on FSD1. HBS: Heparin-binding sequence; FSD, Follistatin/kazal-like domain; EF, a helix-loop-helix structural domain; VWFC, von Willebrand factor type C domain; IGFBP, IGF-binding domain.

Follistatin-like proteins (FSTL) and follistatin (FST) belong to the major family of acidic cysteine-rich secreted glycoproteins that are highly homologous in both primary sequence and domain structure. FSTLs differ in a similar molecular architecture and domain arrangement, and there are five types of FSTLs: FSTL1, FSTL2 (IGFBP7), FSTL3, FSTL4 and FSTL5. These FSTLs contain one or more highly conserved cysteine-rich FST domains. Different FSTLs have their unique physiological functions (Parfenova et al., 2021). FSTL1 is associated with myocardial regenerative processes (Wei et al., 2015), pro-fibrosis (Maksimowski et al., 2021) and promoting inflammation (Wang et al., 2021), and cancer progression (Sundaram et al., 2021; Wu et al., 2021). FSTL2 can bind insulin-like growth factor with high affinity, which can block insulin binding to the insulin receptor (Yamanaka et al., 1997). FSTL4 may be involved in regulating neuronal viability and synapse-related protein expression in primary neurons (Liu et al., 2020). FSTL5 is involved in the perception and processing of olfactory information (Masuda et al., 2014) and may serve as a medulloblastoma prognostic biomarker (Kingwell, 2011). Figure 1 shows the protein domains of FSTLs and FST.

Follistatin-like protein 3 (FSTL3) was first discovered in 1998 by Hayette et al. in a case of B-cell chronic lymphocytic leukemia (Hayette et al., 1998). The FSTL3 gene is localized to human chromosome 19p13 (Phillips and de Kretser, 1998; Maguer-Satta and Rimokh, 2004). The primary sequences of FSTL3 and FST are highly homologous and, therefore, known as the follistatin-related gene (*FLRG*) (Sidis et al., 2002). FSTL3 irreversibly bind with high affinity to transforming growth factor (TGF) -β family ligands, such as activin, bone morphogenetic protein (BMP), and myostatin, rendering them biologically inactive (de Winter et al., 1996) and susceptible to endocytic degradation (Hashimoto et al., 1997). FSTL3 is broadly distributed and highly expressed in the testes, adrenal gland, fetal heart, and placenta. In contrast, FST is maximally expressed in the ovary, pituitary gland, fetal heart, and liver (Tortoriello et al., 2001). Furthermore, FSTL3 knockout mice survive to adulthood and are fertile, whereas the opposite phenotypes are observed in FST knockout mice, highlighting the differential roles of FSTL3 and FST at various developmental stages (Mukherjee et al., 2007). Here, we review the structure and function of FSTL3 in physiology processes, non-tumor pathophysiologies and cancers as well as potential clinical applications targeting FSTL3.

## Structure of FSTL3

Domain organization of FSTLs includes signal peptides and one FST domains (FSD, consisting of a cysteine-rich follistatin module and an evolutionarily conserved Kazal domain). The activin-binding and heparin-binding sites of different FSTLs can locate either before the FSD, or in the N-terminal region, or after it. The C-terminal part contains an immunoglobulin-like domain or C-like domain of von Willebrand factor homologous domain. FSTL3 consists of 263 amino acids, with a molecular weight of 27.6 kDa (Tortoriello et al., 2001). This protein is encoded by five exons, each of which encodes a discrete functional region: a signal peptide, an N-terminal domain (ND), two repeating 10-cysteine (FSD1 and FSD2), and an acidic L-amino acid-containing C-terminal domain (Nakatani et al., 2002) (Figure 1). Although FSTL3 is highly similar to FST, several distinct differences have been observed. First, FST contains an additional exon that encodes FSD3 (Nakatani et al., 2002). Second, FST contains a basic heparin-binding sequence (HBS; residues 75–86), which is absent in the FSD1 region of FSTL3. Therefore, FSTL3 cannot bind to cell surface heparan sulfate proteoglycans (Sidis et al., 2002) and can easily escape into circulation from the cell surface (Ozawa et al., 2021). Finally, FST has two major isoforms, FST288 and FST315. FST303 is the production of the C-terminal tail proteolytic cleavage of FST315, whereas *FSTL3* transcription yields only one transcript (Sidis et al., 2006).

## Function and regulatory mechanisms of FSTL3

Follistatin-like protein 3 (FSTL3) can play an active or inhibitory role in human physiology processes, non-tumor pathophysiologies and cancers by interacting with a disintegrin and metalloproteinase (ADAM) 12, TGF-β ligands and regulates the expression of zyxin, sirtuin-1 (SIRT1), and sclerostin (SOST) (Nam et al., 2015), proteins associated with cell survival, anti-aging, and bone formation and strengthening. FSTL3 acts primarily on TGF-β family ligands, and its binding affinity varies depending on the different specific ligand. FSTL3 has the strongest inhibitory effect on activin, followed by myostatin, and the weakest inhibitory effect on BMPs (Sidis et al., 2006). The differences in binding affinity appear to be tightly correlated with the structural differences in the resulting complexes, especially with regard to the ND. The ND of FSTL3 has a different conformation depending on whether it is bound to myostatin or activin A, and the position of the ND helix changes significantly in both complexes. FSD1 and FSD2 of FSTL3 can block the surface of Activin A type II receptor, and ND can interact with type I receptor interface of Activin A (Stamler et al., 2008), which leads to the dysfunction of Activin A. In contrast to FSTL3 in the activin A complex, the ND in the myostatin complex acts on the wrist region of myostatin by swinging inward from the fingertip. This alters the helical tilt by 21° (Cash et al., 2012). In addition, the substantial hydrophobic interface between the ND of FSTL3 and ligand is crucial for binding. To compensate for the loss of FSD3, the ND of FSTL3 acts on the hydrophobic surface of activin A in a more compact conformation than that of FST (Stamler et al., 2008).

*FSTL3* point mutations L57E and L61E can negatively affect the ligand binding ability of FSTL3, which decreases its inhibitory effect on myostatin compared with wide-type FSTL3. Conversely, the corresponding FST mutations had little effect. BMPs may not be able to accommodate the rigid FSTL3 ND helix at all because of the inflexible structure, which can explain why FSTL3 has little to no affinity for the BMP (Stamler et al., 2008; Cash et al., 2012). As a TGF-β ligand inhibitor, the FSD2 region of FSTL3 bound to iodinated BMP2 and inhibited BMP-2-induced signaling in a rat astrocyte cell line (RNB) (Tsuchida et al., 2000). FSTL3 has also been purified from serum through its binding to myostatin (known as growth differentiation factor (GDF) 8) and was found to inhibit the effect of myostatin (Hill et al., 2002). In addition, FSTL3 can also promote F-actin expression, which results in the change of cytoskeleton and promotes cell motility and proliferation. FSTL3 can act as a bridging molecule between HIPPO/Yes-associated protein 1 (YAP1) and Wnt/β-catenin pathways (Li et al., 2021a). FSTL3 may be a potential binding partner of ADAM12, which is involved in immune infiltration and tumor metastasis in gastric cancer (Zhu et al., 2022). In the hepatocarcinoma HepG2 cell line, activin A was found to increase *FSTL3* transcription through small mothers against decapentaplegic (SMAD) proteins, thereby forming a negative feedback loop (Bartholin et al., 2002). The expression of FSTL3 could be induced by activin A, myostatin and GDF 11 through activin type II receptor induced SMAD2/3 signaling pathway, which showed that circulating FSTL3 can be used as an indirect indication of system pathway activity (Roh et al., 2019). The cooperation of FSTL3 and integrins can regulate the adhesion of hematopoietic cells to fibronectin (Fn), which in turn may play a role in regulating hematopoietic function in humans (Maguer-Satta et al., 2006). The function and regulatory mechanism of FSTL3 in physiologies, various non-tumor pathophysiologies and cancer is complex. The insights gained from studies on function and regulatory mechanism will contribute to the modification of antagonists and their adaptation for potential therapeutic applications.

## The physiological function of FSTL3

FSTL3 expression has been detected in various human and mouse tissues, including steroid glands such as the adrenal gland, testes, ovaries, and plays a critical role in many physiological processes, including energy metabolism, bone formation and strengthening, hematopoietic cell adhesion and hematopoiesis, differentiation of pluripotent stem cells, and reproductive development.

### FSTL3 can affect energy metabolism

Activin A promotes islet cell proliferation and insulin secretion during pregnancy (Petraglia et al., 1995). Myostatin stimulates glucose intake by human full-term villi (Antony et al., 2007) but reduces glucose intake by the human trophoblast cell line BeWo (Mitchell et al., 2006), suggesting that it may regulate glucose homeostasis during pregnancy. As an antagonist of activin A, FSTL3 can play a major role in glucose homeostasis (Mukherjee et al., 2007). FSTL3-null mice exhibit pancreatic β-cell hyperplasia, increased insulin levels, and increased glucose tolerance. Compared to normal pregnancies, women with gestational diabetes mellitus have decreased maternal and placental FSTL3 concentrations, whereas maternal myostatin concentrations remain unchanged. This may implicate FSTL3 in the pathogenesis of gestational diabetes (Hu et al., 2012). FSTL3 is also important for fat metabolism. Knockdown of FSTL3 in rodents causes a reduction in visceral fat mass and leads to increased insulin sensitivity in mice (Mukherjee et al., 2007). FSTL3 levels are increased in patients with nonalcoholic fatty liver disease (NAFLD), with higher FSTL3 levels correlating with increased disease severity. Therefore, FSTL3 may serve as a potential biomarker for the diagnosis of NAFLD (Polyzos et al., 2020).

### FSTL3 promotes bone formation and strengthening

**FIGURE 2:**
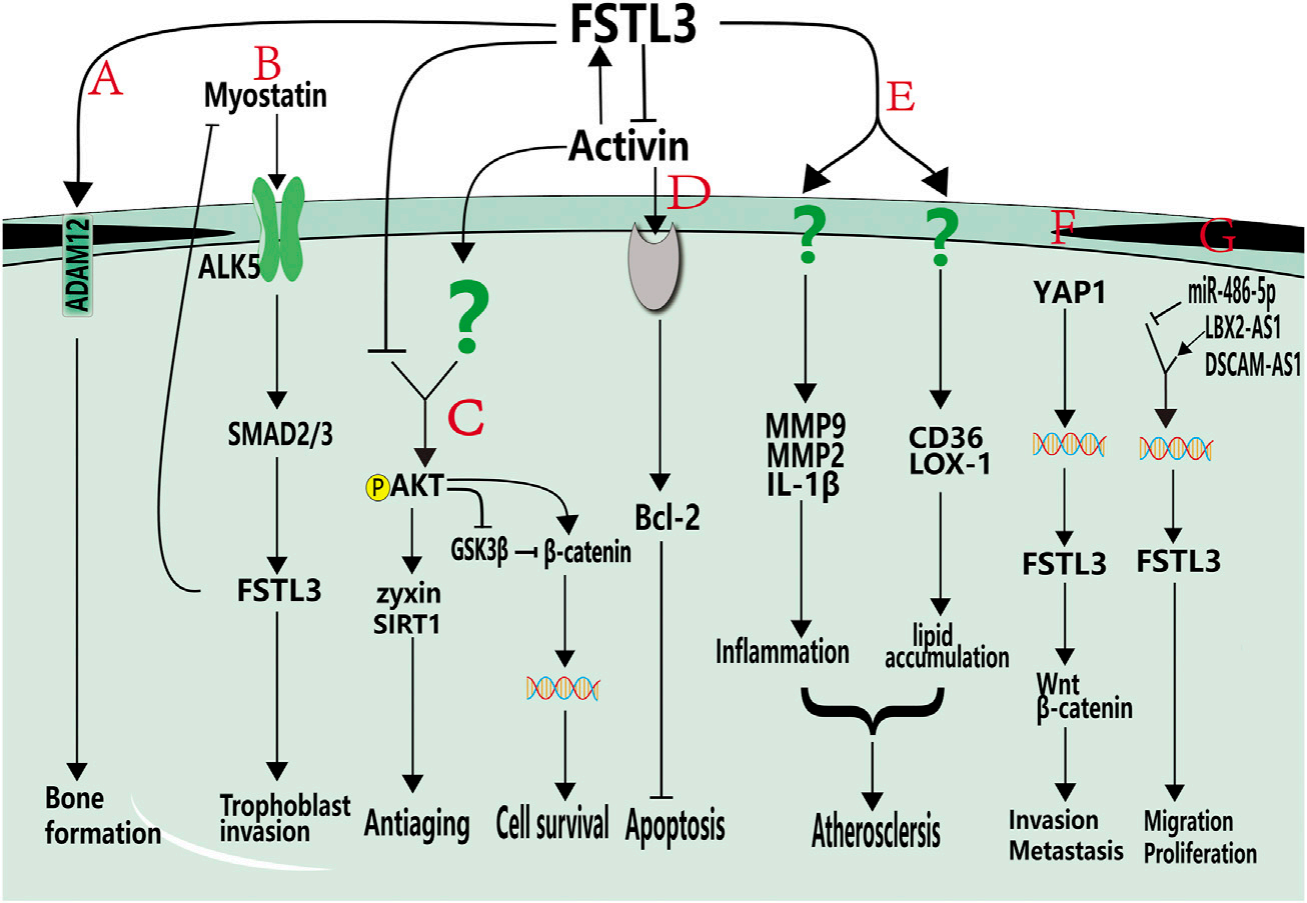
Roles of FSTL3 in non-tumor pathophysiologies and cancers. **(A)**. FSTL3 can bind to ADAM12 and promote bone formation. **(B)**. Myostatin promotes trophoblastic invasion by activating the ALK5-SMAD2/3 signaling pathway and activated SMAD2/3 pathway can increase the expression of FSTL3. **(C)**. FSTL3 inhibits AKT phosphorylation, decreases β-catenin expression, and inhibits cell growth. Additionally, FSTL3 inhibits AKT-mediated activation of zyxin and SIRT1, thereby reducing antiaging effects. **(D)**. FSTL3 inhibits activin A expression, which decreases the expression of Bcl-2. **(E)**. FSTL3 may be induced by oxLDL, upregulates CD36 and LOX-1 and promote MMP9, MCP-1, and IL-1b expression, cause lipid accumulation and inflammatory development, and promotes the development of atherosclerosis. **(F)**. In cancer cells, FSTL3 can serve as a bridging molecule in the crosstalk between YAP1 and β-catenin signaling pathways. The activation of YAP1 can induce FSTL3 expression, and high expression of FSTL3 then activates β-catenin signaling pathways and promotes invasion, migration and metastasis of cancer cells. **(G)**. DSCAM-AS1 and LBX2-AS1 positively regulate the expression of FSTL3 and promote proliferation and migration, and miR-486-5P negatively regulates the expression of FSTL3. Grey components indicate unknown receptors. Colored helical structures represent nuclei. Question marks represent unknown pathways or receptors.

Reproductive system-related proteins can affect bone formation. For example, the reproductive hormones estrogen and androgen are the primary modulators of bone formation, they stimulate bone formation (Venken et al., 2006), prevent osteoporosis (Tivesten et al., 2004), and are vital in bone growth. Similarly, FSTL3 is a reproductive system-associated protein that regulates bone formation. FSTL3 can bind to ADAM12 to reduce the number of osteoclasts and the number of nuclei per osteoclast, inhibit osteoclast differentiation, and promote bone formation (Figure 2A) (Bartholin et al., 2005). Genomic disruption of FSTL3 results in smaller neonatal bone structure without obvious skeletal malformations (Nam et al., 2015). Furthermore, FSTL3 can act as a mediator of exercise-driven bone formation and reinforcement (Nam et al., 2015). Exercise has been reported to upregulate the expression of FST (Hansen et al., 2011) and FSTL1 (Gorgens et al., 2013). Similarly, Nam et al. observed that the expression of FSTL3 was upregulated in musculoskeletal tissues, including bone, cartilage, lateral femoral muscle, and anterior cruciate ligament, of exercised mice compared to that in non-exercised mice (Nam et al., 2015). Circulating FSTL3 level is positively correlated with bone strength (Nam et al., 2015). SOST is a secreted glycoprotein that primarily serves to limit bone formation by inhibiting Wnt signaling (Robling et al., 2008). After 2 h of dynamic compressive strain (DCS) (10%, 0.5 Hz), calvarial osteoblasts from FSTL3 knockout mice exhibited elevated levels of SOST mRNA and protein compared to osteoblasts from control mice. This suggests that FSTL3 may function in a cascade upstream of SOST signaling to regulate bone formation (Nam et al., 2015). In addition, osteocytes isolated from FSTL3 knockout mice did not respond to DCS, indicating that FSTL3 loss can cause mechanical insensitivity. Collectively, these findings indicate that FSTL3 may be a mechanosensitive protein produced in the bone (Nam et al., 2015) that functions in regulating bone formation.

### FSTL3 increases hematopoietic cell adhesion and can inhibit TGF-β family ligands to regulate hematopoiesis

Hematopoiesis is a complex process in which pluripotent stem cells proliferate and differentiate to produce mature blood cells. This process involves complex interactions between soluble factors, including a large number of hematopoiesis-specific cytokines, bone marrow microenvironment, and hematopoietic progenitor cells (Maguer-Satta and Rimokh, 2004). FSTL3 has been found to directly or indirectly affect hematopoiesis. Human Fn was identified as a molecule with strong interactions with FSTL3. FSD1 in FSTL3 is a binding site for the human type I Fn motif. In the presence of FSTL3, adhesion to Fn was significantly increased in the human hematopoietic cell line UT-7, primary cells, and immature hematopoietic cells such as colony-forming cells and long-term culture initiating capabilities cells. It was reduced by adding FSTL3 inhibitors (Maguer-Satta et al., 2006). The role of Fn in the adhesion of human hematopoietic progenitor cells to the bone marrow microenvironment may be a critical factor in regulating hematopoiesis *in vitro* (Verfaillie, 1998). The TGF-β family are important regulators of erythropoiesis; activin A and BMP2 can induce erythropoietin receptor (EPO-R) expression and reduce the expression of endothelial transcription factor GATA-binding factor 2 (GATA-2). Overexpression of GATA-2 inhibits red blood cells and promotes megakaryocyte differentiation (Ikonomi et al., 2000), which is required for red blood cell differentiation. Notably, the highest FSTL3 expression levels were observed in immature erythrocytes. Activin A also induces the expression of EPO-R, hemoglobin, and an inhibitor of cyclin-dependent kinase activity, p27Kip1 (Maguer-Satta et al., 2003). Thus, FSTL3 directly or indirectly controls erythropoiesis by increasing hematopoietic cell adhesion and modulating the effects of activin A and BMP.

### FSTL3 induces the differentiation of pluripotent stem cells

Vascular endothelial cells (ECs) are antithrombotic and maintain hemodynamics, leukocyte activation, adhesion, migration, and vascular homeostasis (Hong et al., 2016). Many functional ECs are required to treat some vascular diseases, necessitating their differentiation from induced pluripotent stem (iPS) cells for clinical use. However, ECs usually exhibit limited proliferative capacity, instability, and senescence; therefore, the ECs generated often achieve limited results in applied treatments (Kruger-Genge et al., 2019). Chen et al. found that Twist1 activates Kruppel-like factor 4 (KLF4) through Jagged1/Notch signaling, induces head and neck cancer cells (HNC) to exhibit stem-like properties, and causes endothelial differentiation (Chen et al., 2014), suggesting that KLF4 may act as a key mediator of ECs differentiation from pluripotent stem cells. FSTL3 is highly expressed in induced Pluripotent Stem Cells (iPS Cells) during the induction of ECs differentiation and is upregulated by KLF4 (Kelaini et al., 2018). FSTL3 has been speculated to play an important role in maintaining the generation of iPS-ECs. In the iPS-induced ECs model, FSTL3 expression increased with increasing KLF4 levels. FSTL3 was positively regulated by KLF4 and played a significant role in promoting iPS-ECs generation (Kelaini et al., 2018).

FSTL3-induced expression of endothelin-1 is also critical for iPS-ECs generation (Kelaini et al., 2018). Endothelin-1 induces angiogenic responses in endothelial cells and is the most highly expressed angiogenic factor detected in iPS-ECs using an angiogenesis analyzer array (Salani et al., 2000). Endothelin-1 inhibits the activity of GSK3β by increasing its phosphorylation. In iPS-ECs, FSTL3 was associated with endothelin-1 expression, which led to GSK3β inactivation. This, in turn, enhanced β-catenin nuclear translocation, thereby increasing the expression of the ECs markers CD34, KDR, CD31, CD144, eNOS, and vWF (Kelaini et al., 2018). Therefore, FSTL3 is an important factor in differentiating human iPS Cells into ECs.

### The function of FSTL3 in reproductive development

In the reproductive system, the interaction of FSTL3 and myostatin can promote the invasion of trophoblast cells, and FSTL3 involves in cell aging and survival through regulating the phosphorylation of AKT. FSTL3 can promote adhesion and infiltration of trophoblast cells. Trophoblast cells differentiate into villous trophoblasts and extravillous trophoblasts (EVTs) after early embryo implantation in the endometrium. During normal pregnancy, EVTs play an important role in embryo implantation and fetal development. EVTs can actively proliferate, adhere to, and infiltrate the maternal uterine wall; and participate in uterine spiral artery remodeling. Uncontrolled proliferation and invasion by EVTs can cause various trophoblast cell-related neoplastic diseases.

In particular, FSTL3 can promote adhesion and infiltration of trophoblast cells, which is associated with preeclampsia development (Biron-Shental et al., 2008; Xie et al., 2018; Xie et al., 2020). Preeclampsia is caused by insufficient trophoblast invasion, leading to failure of uterine spiral artery remodeling (Williamson et al., 2015). It is one of the most serious pregnancy-related diseases and involves the liver, kidney, lungs, and potential multi-organ failure; and can result in coagulation and nervous system damage (Duley, 2009). Therefore, exploring the mechanisms of trophoblast adhesion and infiltration in preeclampsia could lead to the development of a potential treatment. FSTL3 is highly expressed in patients with preeclampsia and positively regulates trophoblast function. Hypoxia is a characteristic feature of preeclampsia; trophoblasts highly express FSTL3 after using hypoxia-mimetic agents, and hypoxia-inducible factor-1 alpha (HIF1α) may be involved in promoting FSTL3 expression (Biron-Shental et al., 2008). Xie et al. observed that downregulating FSTL3 inhibited the proliferation, migration, invasion, and lipid storage capacity of trophoblasts while promoting apoptosis, indicating that FSTL3 can promote trophoblast proliferation (Xie et al., 2018). The TGF-β family member, myostatin, is highly expressed in patients with preeclampsia. The dynamic change in myostatin is more than that in FSTL3 in women with preeclampsia. Antagonism of myostatin by FSTL3 may not affect the physiological function of myostatin. SMAD2/3 activation induces *FSTL3* transcription, which regulates the trophoblast invasion. FSTL3 activation is necessary to inhibit continued myostatin action (Figure 2B) (Xie et al., 2020). Matrix metalloproteinase 9 (MMP9) is strongly expressed in trophoblasts during early pregnancy and can regulate trophoblast invasion (Zhu et al., 2012). FSTL3 can promote the expression of MMP9, CD36, and IL-1β (Runhua et al., 2019). However, Jiang et al. reported that the inhibition of FSTL3 could improve the migration and invasion of hypoxic trophoblast cells, with increased viability, angiogenesis, and inflammatory response. They observed that human umbilical cord mesenchymal stem cell-released exosomes exerted a protective effect on preeclampsia by mediating the transmission of miR-140-5p, primarily by targeting FSTL3. Inhibition of FSTL3 by miR-140-5p accelerates the growth of hypoxic trophoblast cells; therefore, the role of FSTL3 in trophoblast cells must be explored further (Jiang et al., 2022).

FSTL3 not only plays a regulatory role in the female reproductive system, but also has a non-negligible role in spermatogenesis and testicular degeneration in men. FSTL3 can regulate spermatogenesis through AKT activation. In normal mouse testes, endogenous FSTL3 is expressed in mature spermatids, spermatogonia at the base of tubules, and in Leydig cells (Xia et al., 2004). In FSTL3 knockout mice, the testes were structurally unchanged, but increased proliferation of Sertoli cells and associated germ cells and increased testicular volume were observed (Lee et al., 2004). The absence of FSTL3 could increase activin action and activate AKT-dependent signaling (Oldknow et al., 2013). AKT phosphorylation inactivates glycogen synthase kinase 3 beta (GSK3β), which mediates the maintenance of β-catenin to promote cell survival (Figure 2C). This study also suggested that the testicular degeneration was delayed and did not change with increasing age. The TUNEL assay results showed reduced spermatogonia apoptosis. Western blotting analysis revealed that the expression of zyxin and SIRT1 proteins was upregulated in the testes of FSTL3 knockout mice compared to that in control mice. Therefore, the effects of FSTL3 on mouse testes may be involved in AKT-mediated phosphorylation of zyxin (Lee et al., 2004) and the anti-aging effects caused by the interaction of zyxin with SIRT1 (Figure 2C) (Cohen et al., 2004; Oldknow et al., 2013). In contrast, transgenic mice overexpressing FSTL3 had smaller gonads than their wild-type littermates. FSTL3 overexpression in testes could result in smaller testes, arrest of spermatogenesis, tubule degeneration, and Leydig cell hyperplasia (Xia et al., 2004). In humans, defects in Sertoli cell maturation are the main cause of male infertility (Sharpe et al., 2003). Therefore, FSTL3 may play a key role in regulating Sertoli cell number and spermatogenesis and has the potential to be a useful therapeutic approach for the treatment of male infertility caused by insufficient sperm production.

## Role of FSTL3 in various non-cancerous diseases

In addition to its role in human physiology processes, FSTL3 is also a crucial regulatory factor in the cardiac hypertrophy and myocardial fibrosis, atherosclerosis, *via* regulating the expression of other proteins including activin A, myostain, Bcl-2.

### FSTL3 is implicated in cardiac hypertrophy and myocardial fibrosis

Diseases such as peripheral arterial hypertension led to increased cardiac pumping resistance. The cardiac pathological response to sustained pressure overload involves myocyte hypertrophy and dysfunction, as well as interstitial changes (Koitabashi et al., 2011). FSTL3 is highly expressed in cardiomyocytes and endothelial cells and modulates stress-induced cardiac hypertrophy. After pressure overload induced by transverse aortic constriction, FSTL3 knockout mice exhibited diminished cardiac hypertrophy, reduced left ventricular wall thickness, and left ventricular dilatation. This may be mediated by the upregulation of activin A following FSTL3 knockdown and protects cardiomyocytes from injury by altering the expression of the anti-apoptotic protein Bcl-2 (Figure 2D) (Oshima et al., 2009). In addition, the enhanced SMAD2 phosphorylation (Shimano et al., 2011) exerted an inhibitory effect on cardiac hypertrophy. These findings implicate FSTL3 as a stress-induced regulator of hypertrophy that controls myocyte size by regulating SMAD signaling (Shimano et al., 2011).

FSTL3 can regulate myocardial fibrotic change and affect myocardial hypertrophy. Elevated myocardial stress can trigger the structural remodeling of the heart. A sustained increase in wall stress can exceed the compensatory capacity of the heart, causing the ventricular geometry to become spherical, significant myocardial fibrosis, and eventually heart failure (Brower et al., 2006). The cardiac structure is maintained by the extracellular matrix (ECM), which supports the normal contractile heart function. The cardiac ECM contains fibrillar collagen, Fn, glycoproteins, and proteoglycans (Frangogiannis, 2019). FSTL3 increases Fn adhesion (Maguer-Satta et al., 2006). FSTL3 produced by cardiomyocytes promotes the paracrine activation of cardiac fibroblasts, inducing changes in cell adhesion, promoting proliferation and increasing collagen production (Panse et al., 2012). Myocardial fibrosis is attenuated in FSTL3 knockout mice (Shimano et al., 2011). Inhibiting FSTL3 by neutralizing antibodies may be a potential therapeutic strategy for minimizing its deleterious effects on cardiomyocytes and fibroblasts, thereby inhibiting the development of cardiac hypertrophy and myocardial fibrosis (Bartholin et al., 2002; Panse et al., 2012). Alternatively, FSTL3 expression levels could be used as a biomarker for heart failure. Elevated levels of FSTL3 have been detected in the myocardium of patients with heart failure. The expression levels of FSTL3 positively correlated with α-skeletal actin and brain natriuretic peptide, both markers of heart failure severity (Lara-Pezzi et al., 2008). After left ventricular assist device implantation for heart failure, FSTL3 levels returned to normal (Lara-Pezzi et al., 2008).

### FSTL3 promotes atherosclerosis

Atherosclerosis, a chronic inflammatory disease of the blood vessels, can cause stroke, ischemic heart disease, and peripheral vascular disease (Kobiyama and Ley, 2018). It is mainly caused by the deposition of oxidized low-density lipoproteins (oxLDL) into the intima leading to the recruitment of macrophage T cells and other immunoreactive cells (Hansson and Hermansson, 2011). Histological analysis of patients with atherosclerosis revealed elevated FSTL3 expression. In Apo-E knockout mice atherosclerotic plaques, oxLDL can induce the expression and secretion of FSTL3 in macrophages (Runhua et al., 2019), which led to the upregulation of CD36 and lectin-like oxLDL receptor-1 (LOX-1). As a recognition receptor, CD36 interacts with oxLDL, recruits macrophages to the intima, inhibits macrophage migration, and plays an important role in atherosclerosis (Tian et al., 2020). Therefore, FSTL3 is linked to atherosclerosis by its role in upregulating CD36 and promoting lipid accumulation in macrophages (Figure 2E) (Runhua et al., 2019).

In addition to its ability to promote macrophage lipid accumulation, FSTL3 is also crucial in inflammation regulation. Obesity and inflammation can promote the release of FSTL3 (Brandt et al., 2014). Tumor necrosis factor alpha (TNF-α), which is upregulated during atherosclerosis (Runhua et al., 2019), promotes *FSTL3* transcription by binding to four TNF-α sensitive repeats in its promoter region (Bartholin et al., 2007). FSTL3 also induces macrophages to secrete monocyte chemoattractant protein-1 (MCP-1), interleukin-1b (IL-1b), and MMP9 (Runhua et al., 2019), which are involved in the induction of arterial changes. In summary, FSTL3 signaling contributes to the pathogenesis of atherosclerosis after LDL oxidation by regulating lipid accumulation and inflammation (Figure 2E).

## Role of FSTL3 in cancers

FSTL3 was first reported in a case of B chronic lymphocytic leukemia but was not found in normal B lymphocytic bone marrow cells. Since leukemic FSTL3 overexpression may result from genetic alterations, FSTL3 may be involved in the tumorigenic process (Hayette et al., 1998). In recent years, studies have found that FSTL3 expression is elevated in most tumors and is associated with poor prognosis. FSTL3 is often associated with the progression of malignant tumors by inducing the epithelial-mesenchymal transition (EMT), affecting the tumor microenvironment, influencing non-coding RNA regulation, and promoting angiogenesis. Therefore, FSTL3 has the potential to be used as a tumor-related marker to improve clinical decision-making.

### FSTL3 promotes metastasis

Tumor metastasis refers to the process by which tumor cells move from the primary tumor site to colonize distant tissues and organs and establish new tumors (Valastyan and Weinberg, 2011). Tumor metastasis is often closely related to neovascularization (Folkman, 2002), EMT (Yeung and Yang, 2017), and the secretion of MMPs (Ray and Stetler-Stevenson, 1994). EMT is essential for embryonic development and tissue repair, and is characterized by reduced expression of E-cadherin and increased expression of the mesenchymal markers, vimentin, N-cadherin, and Fn. These changes lead to enhanced cell motility and cytoskeletal remodeling, which facilitates migration and invasion.

High expression level of FSTL3 is closely associated with the strong abilities of migration and invasion of cancer cells. FSTL3 expression is correlated with lymph node metastasis, tumor stage, tumor size, and intravascular emboli and is associated with a poorer prognosis (Li et al., 2021a; Yang et al., 2021). Studies have reported EMT changes in tumor cells exposed to short hairpin RNAs targeting FSTL3 (shFSTL3) in the human colon cancer cell line HCT116 (Li et al., 2021a). The reduction of FSTL3 levels enhanced E-cadherin expression while decreasing the expression levels of β-catenin, N-cadherin, and vimentin, as well as reduced F-actin polymerization in the cells. In the same study, FSTL3 overexpression increased the expression of proteins involved in aerobic glycolysis, such as GLUT1, LDHA, HK2, and PKM2, resulting in a decrease in the pH of the tumor microenvironment and contributing to tumor cell immune evasion (Li et al., 2021a). Li et al. investigated the mechanism by which FSTL3 promotes the invasion and metastasis of colorectal cancer cells. This study revealed that FSTL3 might serve as a bridging molecule that mediates the crosstalk between the HIPPO/YAP1 and Wnt/β-catenin pathways (Figure 2F) (Li et al., 2021a). The activation of YAP1 induced FSTL3 expression, which in turn activated the β-catenin pathway. β-catenin can induce YAP1. YAP1 can transactivate FSTL3, thereby forming a positive feedback loop that promotes EMT, aerobic glycolysis, and the invasion and metastasis of colorectal cancer cells (Li et al., 2021a). FSTL3 is also involved in the dysregulation of other cancer stem cells; for example, FSTL3 facilitates the proliferation, invasion, and drug resistance of glioblastoma multiforme stem cells (Du et al., 2020).

In addition to its role in colorectal cancer, FSTL3 is also overexpressed in human gastric cancer cells and promotes their invasion of other tissues. Previous work demonstrated that overexpression of FSTL3 promotes F-actin expression. Result of gene set enrichment analysis suggested that FSTL3 interacted with lamellipodia, filamentous actin, invadopodia, gamma-tubulin, and activated EMT (Liu et al., 2021a). FSTL3 and ADAM12 have similar functions to regulate tumor metastasis and immune infiltration in gastric cancer (Zhu et al., 2022).

### FSTL3 is a potential regulator of angiogenesis

The expression level of FSTL3 is also associated with tumor angiogenesis. FSTL3 is highly expressed in the blood vessels of granulation tissue, and its expression can be induced by keratinocyte growth factor, TGF-β, and epidermal growth factor (Wankell et al., 2001). In endometrial cancer, immunohistochemical staining revealed that FSTL3 was feebly expressed in endometrial tumor glands but strongly expressed in the surrounding capillaries (Ciarmela et al., 2004). In non-small cell lung cancer (NSCLC), FSTL3 has been found to be closely associated with angiogenic factors based on bioinformatics studies and may serve as a potential anti-angiogenic therapeutic target (Gu et al., 2022). Activin A can inhibit vascular endothelial cell growth (McCarthy and Bicknell, 1993), and FSTL3 enhances the vascular proliferation and angiogenesis by inhibiting the expression of activin A (Ciarmela et al., 2004).

### FSTL3 is regulated by non-coding RNAs in malignant tumors

Non-coding RNAs (ncRNAs) are RNAs that do not encode proteins but can regulate biological processes to drive specific cellular responses. These include microRNAs (miRNAs), long non-coding RNAs (lncRNAs), and circular RNAs. NcRNAs were found to regulate the expression of FSTL3, thereby affecting cancer cell genesis. miRNAs are small, endogenously expressed ncRNAs, 18–25 nucleotides in length, regulating mRNA degradation and/or translational repression by binding to the 3′-untranslated region (3′-UTR) of target mRNAs (Bartel, 2004). The dysregulation of miRNAs has been implicated in cancer biology; lower miR-486-5p levels and higher FSTL3 expression have been found in gastric cells of patients with gastric cancer compared to that in healthy controls (Dai et al., 2021). *In silico* analysis predicted that miR-486-5p binds to the 3′-UTR of FSTL3 mRNA and decreases its mRNA and protein levels. Knockdown of FSTL3 by shFSTL3 was rescued by adding a miR486-5p inhibitor, confirming that FSTL3 mRNA is negatively regulated by miR-486-5p. These data support a role for miR-486-5p in regulating FSTL3 expression and gastric cancer cell responses such as cell viability, proliferation, and migration (Figure 2G) (Dai et al., 2021).

LncRNAs are a class of non-coding RNAs involved in gene regulation. They are coactivators that bind to transcription factors and enhance their transcriptional activity (Anastasiadou et al., 2018) and can regulate FSTL3 expression in malignant tumors. The lncRNA LBX2-AS1 was significantly enriched in the thyroid tissues of patients with thyroid cancer than in healthy thyroid tissues. Following transfection of tumor tissues from mice with shLBX2-AS1, western blotting results showed that the protein levels of FSTL3, vimentin, N-cadherin, MMP2, and MMP9 decreased. Additionally, immunohistochemistry revealed a decrease in the proliferation marker, Ki67. These results indicate that LBX2-AS1 promotes the malignant migration and invasion of thyroid cancer cells. FSTL3 can be implicated in these phenotypes *via* the nuclear receptor transcription factor retinoic acid receptor (RARα), which can interact with target genes to regulate normal physiological processes, tumor growth, metastasis, drug resistance, and other processes (Khetchoumian et al., 2008). LBX2-AS1 upregulates the expression of FSTL3 by recruiting RARα to the FSTL3 promoter, which suggests that FSTL3 may play a role in accelerating the progression of thyroid cancer (Figure 2G) (Li et al., 2021b). In NSCLC, high expression of FSTL3 is significantly correlated with lymph node invasion (Gao et al., 2020) and abnormally high expression of the lncRNA down syndrome cell adhesion molecule antisense 1 (DSCAM-AS1). DSCAM-AS1 promotes breast cancer development (Liang et al., 2019). Additionally, miR-122-5p is expressed at low levels in NSCLC cells and negatively regulates the expression of FSTL3, which may regulate EMT in these cells. In NSCLC, DSCAM-AS1 participates in a competitive endogenous mechanism: DSCAM-AS1 indirectly upregulates FSTL3 expression by acting as a competing endogenous RNA against miR-122-5p, thereby accelerating the development and progression of NSCLC (Figure 2G) (Gao et al., 2020).

## Potential clinical applications of small molecule inhibitors and neutralizing antibodies targeting FSTL3

Current studies about FSTL3 have drawn some meaningful conclusions. High FSTL3 expression is associated with high malignancy in colorectal, gastric, thyroid, NSCLC, and renal cell carcinoma, which suggest that FSTL3 may be a promising target in the clinical treatment of malignant tumors. Inhibitors and neutralizing antibodies against FSTL3 have been used in the treatment of diabetes and muscle wasting *in vivo*.

Decreased β-cell function and insulin resistance become difficult therapeutic problems in type 2 diabetes (Cohrs et al., 2020). Previous studies proved that enlarged islets could be observed in FSTL3 KO mice. The enlarged islets contain more β-cell numbers, secret more insulin, and enhance glucose tolerance (Mukherjee et al., 2007). Further studies demonstrated that activin could enhance the transdifferentiation of α to β cells in FSTL3 knockout mice (Brown et al., 2016). Brown et al. identified that a FSTL3 neutralizing antibody, FP-101 (Brown et al., 2021), could effectively prevent FSTL3 to complex with activin or related ligands, and inhibit the release of activin from the nearly irreversible FSTL3-activin complex. The antibody is selective for FSTL3 and does not inhibit FST, a highly structural homologue of FSTL3, thereby reducing off-target effects. *In vitro* isolated islets experiments have demonstrated that FP-101 could enhance insulin secretion.

Neutralizing antibodies to FSTL3 also show potential clinical application in the treatment of diabetes and muscle wasting. Myostatin can inhibit normal muscle growth, and targeting myostatin may be used as a promising strategy to increase muscle mass in elderly or patients with muscle atrophy (Cohen et al., 2015). The clinical trial of ACE-031 (myostatin inhibitor) was discontinued because of its broad binding to BMP9, BMP10, and other angiogenic TGF-b family members, with a risk of bleeding (Campbell et al., 2017). Monovalent FSTL3-Fc-fusion protein could increases muscle mass in muscle wasting and neuromuscular diseases (Ozawa et al., 2021).

Small molecule inhibitors targeting YAP1 can also reduce FSTL3 expression. In prostate cancer, YAP1 expression is positively correlated with FSTL (Lee et al., 2017). Li et al. reported that pharmacological targeting of YAP1 could counteract FSTL3 expression and may serve as a therapeutic target for CRC patients (Li et al., 2021a). Simvastatin, an inhibitor of YAP1, can inhibit the biological activity of YAP, increase the sensitivity of CRC cells to EGFR inhibitors, and improve therapeutic efficacy (Lee et al., 2015). Regorafenib can also inhibit YAP1 activity in cholangiocarcinoma cells, and regorafenib combined with amphiregulin neutralize antibody significantly decrease metastatic phenotype and EMT *in vitro* and *in vivo* (Chang et al., 2022). In summary, FSTL3 plays an important role in human non-tumor pathophysiologies and cancers, and may be targets of the potential clinical therapy. Studies about the regulatory mechanism and function of FSTL3 may provide new therapeutic strategies for cancer and other pathologies.

## Conclusion

As a member of the FST family, FSTL3 has been proved to play a critical role in human physiological process, non-tumor pathophysiologies and cancer. FSTL3 not only increase myocardial fibrosis (Panse et al., 2012), promote bone formation and strengthening (Bartholin et al., 2005), regulates the reproductive system, but also can enhance the migration and invasion abilities of a variety of malignant tumor cells by increasing glycolysis, decreasing PH in the tumor microenvironment and resulting in EMT of tumor cells, which is closely associated with the poor prognosis of patients (Razanajaona et al., 2007; Li et al., 2021a). In this review, we describe the potential molecular mechanisms underlying the action of FSTL3. The role of FSTL3 in non-tumor pathophysiologies and cancer was summarized in Tables 1, 2. However, the intrinsic detailed mechanism of FSTL3 in human non-tumor pathophysiologies and cancer requires further study. Understanding the role of FSTL3 in human non-tumor pathophysiologies and cancer may provide new ideas for exploring therapeutic targets for human diseases.

**TABLE 1:** FSTL3 in non-tumor pathophysiologies.

Non-tumor pathophysiologies	Function and mechanisms of FSTL3	Ref
Cardiac hypertrophy	As TGF-β ligand inhibition, FSTL3 knockdown negatively regulates activin expression, enhances downstream SMAD2 phosphorylation, and increases Bcl-2 expression, exerting a role in protecting cardiomyocytes and inhibiting cardiac hypertrophy	Shimano et al. (2011)
Spermatogenesis	Increased activin expression after FSTL3 deletion may active AKT-dependent signaling. AKT phosphorylation inhibits GSK3β, increases β-catenin expression, activates zyxin, and binds SIRT1, exerting anti-aging effects	Lee et al. (2004)
Bone formation	FSTL3 can bind to ADAM12, reduce osteoclast number and average nuclear number per osteoclast, inhibit osteoclast differentiation, and promote bone formation. In addition, FSTL3 is associated with bone formation by regulating SOST expression	(Bartholin et al., 2005), (Nam et al., 2015)
Differentiation of pluripotent stem cells	FSTL3 regulated by KLF4 can enhance EC features by facilitating β-catenin nuclear translocation through inhibition of GSK3β activity and induction of endothelian-1 in iPS cells	Kelaini et al. (2018)
Atherosclerosis	FSTL3 is associated with atherosclerosis by up-regulating CD36 and promoting lipid accumulation in macrophages	Runhua et al. (2019)
Preeclampsia	SMAD2/3 activation induces *FSTL3* transcription, which regulates the trophoblast invasion. FSTL3 activation is necessary to inhibit continued myostatin action	Xie et al. (2020)
Heart failure	FSTL3 expression levels may serve as biomarkers of heart failure	Lara-Pezzi et al. (2008)
NAFLD	FSTL3 may serve as a potential biomarker for the diagnosis of NAFLD.	Polyzos et al. (2020)

**TABLE 2:** Function of FSTL3 in different types of cancer.

Tumor	Function and mechanisms of FSTL3	Ref
Gastric cancer	FSTL3 may be a potential binding partner of ADAM12. FSTL3 and ADAM12 perform similar functions to regulate tumor metastasis and immune infiltration in gastric cancer. In addition, FSTL3 overexpression promotes F-actin and BMP/SMAD signaling to activate EMT and M2 macrophage activation	Zhu et al. (2022), Liu et al. (2021a)
Colorectal cancer	FSTL3 can act as a bridging molecule for the crosstalk between HIPPO/YAP1 and Wnt/β-catenin pathways. Activation of YAP1 induces FSTL3 expression, which in turn activates the β-catenin pathway. β-catenin can induce YAP1, which trans-activates FSTL3, thereby forming a positive feedback loop and promoting EMT, aerobic glycolysis, invasion and metastasis of colorectal cancer cells. In addition, FSTL3 is associated with chemoresistance in lymph node metastasis and can be used as a marker of poor prognosis	Li et al. (2021a) Liu et al. (2021b), Yang et al. (2021)
NSCLC	LncRNA DSCAM-AS1 and miR-122-5P can elevate FSTL3 expression and promote the proliferation and migration of NSCLC.	Gao et al. (2020)
Renal cell carcinoma	FSTL3 enhances the proliferation and metastasis of renal cell carcinoma through the GSK3β/β-catenin signaling pathway	Sun et al. (2021)
Thyroid cancer	LBX2-AS1 promotes thyroid cancer development by recruiting RARα to regulate FSTL3 expression	Li et al. (2021b)
Breast cancer	FSTL3 can be used to differentiate breast cancer from benign lesions	Panagiotou et al. (2019)

## Glossary

ADAM12: a disintegrin and metalloproteinase 12
BMP: bone morphogenetic protein
DCS: dynamic compressive strain
EMT: epithelial-mesenchymal transition
EVTs: extravillous trophoblasts
ECM: extracellular matrix
EPO-R: erythropoietin receptor
ECs: endothelial cells
FLRG: follistatin related gene
FSD: follistatin domains
FST: follistatin
FSTL: follistatin-like protein
FSTL3: follistatin-like protein 3
Fn: fibronectin
GATA-2: endothelial transcription factor GATA-2
GDF: growth differentiation factor
GSK3β: glycogen synthase kinase 3 beta
iPS Cells: induced Pluripotent Stem Cells
IL-1b: interleukin 1-b
KLF4: kruppel-like factor 4
LOX-1: lectin-like oxLDL receptor-1
lncRNAs: long non-coding RNAs
MCP-1: monocyte chemoattractant protein-1
MMP2: matrix metalloproteinase 2
MMP9: matrix metalloproteinase 9
miRNAs: microRNAs
mRNA: messenger RNA
NAFLD: nonalcoholic fatty liver disease
NSCLC: non-small cell lung cancer
oxLDL: oxidized low-density lipoproteins
RARα: retinoic acid receptor alpha
SOST: sclerostin
SMAD: small mothers against decapentaplegic
SIRT1: sirtuin-1
TGF-β: transforming growth factor-β
TNF-α: tumor necrosis factor alpha
YAP1: Yes-associated protein 1
